# Eco-hydrological modelling of channel network dynamics—part 1: stochastic simulation of active stream expansion and retraction

**DOI:** 10.1098/rsos.220944

**Published:** 2022-11-16

**Authors:** Nicola Durighetto, Leonardo E. Bertassello, Gianluca Botter

**Affiliations:** ^1^ Department of Civil, Environmental and Architectural Engineering, University of Padua, via Loredan 20, Padova 35131, Italy; ^2^ University of Notre Dame, Notre Dame, IN 46556, USA; ^3^ Dipartimento di ingegneria civile edile, Università degli Studi di Padova, ambientale e architettura, Padova 35131, Italy

**Keywords:** non-perennial streams, temporary streams, network dynamics, stochastic modelling, streamflow, active length

## Abstract

Dynamic changes in the active portion of stream networks represent a phenomenon common to diverse climates and geologic settings. However, mechanistically describing these processes at the relevant spatiotemporal scales without huge computational burdens remains challenging. Here, we present a novel stochastic framework for the effective simulation of channel network dynamics capitalizing on the concept of ‘hierarchical structuring of temporary streams’—a general principle to identify the activation/deactivation order of network nodes. The framework allows the long-term description of event-based changes of the river network configuration starting from widely available climatic data (mainly rainfall and evapotranspiration). Our results indicate that climate strongly controls temporal variations of the active length, influencing not only the preferential configuration of the active channels but also the speed of network retraction during drying. Moreover, we observed that—while the statistics of wet length are mainly dictated by the underlying climatic conditions—the spatial patterns of active reaches and the size of the largest connected patch of the network are strongly controlled by the spatial correlation of local persistency. The proposed framework provides a robust mathematical set-up for analysing the multi-faceted ecological legacies of channel network dynamics, as discussed in a companion paper.

## 1. Introduction

The flowing portion of many river networks does vary in time, owing to seasonal and/or event-based expansion–retraction cycles that mimic the unsteady nature of the underlying climatic forcing. Such rivers, commonly referred to as temporary streams, are believed to represent more than half of the global river network and are observed in most climatic regions worldwide [1–3]. The dynamic nature of channel networks has important implications beyond catchment hydrology, which include nutrient cycling, greenhouse gas emissions, stream metabolism, ecological dispersion and water management [1,4–13]. Quantifying the impact of stream dynamics on ecological and biochemical processes, however, requires a proper characterization of the expansion/retraction cycles experienced by stream networks in response to the ever-changing hydrological conditions of the surrounding landscape [14].

The first hydrologic studies about temporary streams date back to half a century ago [15–23]. Those pioneering works revealed the twofold challenge that underlies the study of temporary streams: while collecting empirical data requires a significant experimental burden, extensive datasets are necessary to disentangle the intertwined spatial and temporal dimensions of the problem, which complicate the identification of the physical processes underpinning the activation/deactivation of different stream portions. Even though field monitoring remains labour intensive, the last decade has seen a significant increase in the number of available datasets. These datasets, however, seldom span more than a few years and are mostly characterized by coarse (e.g. biweekly) temporal resolutions [3,24–33].

To explain the main drivers of network dynamics, [30,34] proposed a conceptual model that links the surface flow at a point to the imbalance between the downvalley seepage rate and subsurface transmissivity. However, the application of this conceptual model for the prediction or the simulation of the spatial patterns of the flowing streams is problematic, as it would require the specification of spatial patterns of subsurface transmissivity and valley cross-sectional area, which are very difficult to measure or predict.

In recent years, there have been few practical attempts to reproduce the full spatial and temporal dynamics of the actively flowing channels within a river network: (i) [35] used a detailed, physically based, distributed model that simulated surface water–groundwater interactions and active length variations along a 2.9 km channel in the western Cascades, Oregon, USA; (ii) [36] created a logistic regression model that combined catchment discharge measurements and spatial patterns of morphometric attributes, which can be potentially used to simulate network dynamics; and (iii) [29,37] employed a statistical approach in which the dynamics of the active length were linked to climatic attributes (antecedent rainfall, evapotranspiration). Therein, the spatial patterns of channel activations were either specified based on high-frequency observations or predicted based on geological and lithological properties. All these modelling attempts were extremely valuable in clarifying the major climatic and geologic determinants of stream network expansion/retraction. However, the aforementioned models can hardly be generalized and used for predictive purposes outside the specific context in which they were conceived, in particular for applications to study sites where empirical data on network dynamics are lacking. Moreover, the existing literature lacks stochastic approaches that are capable of linking the spatial and temporal dynamics of the active portion of the river network to the underlying rainfall and streamflow regimes. This emphasizes the need for developing general but parsimonious models—potentially applicable even to ungauged locations—for the synthetic generation of long-term scenarios representative of different hydroclimatic regimes.

In the following, we first present a novel model for the stochastic simulation of the dynamics of the flowing network (§§2.1 and 2.2). The proposed model, which relies on a small number of parameters and requires a limited computational effort, allows the synthetic simulation of the spatiotemporal dynamics of the active stream network under a broad range of hydroclimatic conditions (§2.3). The model is used to explore spatiotemporal dynamics of the active network in streams subjected to contrasting flow regimes and various spatial patterns of flow persistency (§3.1).

A discussion of the relevance of our findings (§4) and a set of conclusions (§5) then close the paper.

The potential of the tool will be further demonstrated in a companion paper, where the proposed hydrological model will be coupled to a dynamic version of a stochastic ecological model to explore the effect of stream expansion and retraction on the survival probability of a representative aquatic animal species.

## 2. Methods

A dynamic stream network is here represented by a set of *N* nodes with arbitrary spatial coordinates and a binary state. Each node (*i*) is representative of the hydrological conditions in a uniform stream reach of length Δ*l*_*i*_ containing the node *i*. At any time, each node has a status *X*_*i*_(*t*) that could be either 0 (dry) or 1 (active). Temporal changes in the spatial configuration of the active network are simulated by assigning the status of each node in the network during a sequence of time steps. The time variability of the status of each node can be summarized by its local persistency, *P*_*i*_, which represents the marginal probability of node *i* being active. The spatial correlation among the status of each node, inherent to real-world stream network dynamics, is set using the persistency-driven hierarchical behaviour proposed by [14,38], according to which the nodes are always activated from the most to the least persistent during network expansion and deactivated in the reverse order during retraction (note that *P*_*i*_ may vary non-monotonically in space, thereby generating disconnections along the active network). This corresponds to a stochastic process in which nodes are ordered in a chain with decreasing local persistency and pairwise links are set between consecutive nodes. The status of each node is then conceived as a random variable conditionally dependent on the status of the previous node in the chain. The (spatially variable) conditional probabilities of the states of the nodes are determined in line with what is prescribed by the hierarchical behaviour itself: the model prescribes that node *i* can be active only if all previous nodes in the chain (i.e. all the nodes with larger local persistency) are active too. As a consequence, each possible network configuration corresponds to a sequence of *K* active nodes (*K* ∈ [1, *N*]) followed by a sequence of *N* − *K* dry nodes, and a biunivocal relation between the active length and its spatial configuration along the network is generated. This is tantamount to assuming that at any time there exists a time-dependent persistency threshold P*(*t*) that separates dry nodes (the nodes with persistency lower than P*(*t*)) from wet nodes (the ones with persistency higher than P*(*t*)). The stochastic variability of P*(*t*) surrogates in a simplified manner the probabilistic nature of the underlying hierarchical network. The hierarchical mechanism implies that nodes with the same persistency display a synchronized behaviour even if they are not close-by in the physical space. It is important to stress that this hierarchical approach can also reproduce disconnected networks: local disconnections are in fact observed wherever a low-persistency node is embedded within a more persistent reach, while disconnected wet segments are observed when nodes with a higher persistency are hosted within more ephemeral reaches. The advantage offered by this approach is the disentanglement of the temporal dimension of network dynamics, dictated by P*(*t*), from the spatial pattern of the active nodes, which is prescribed by the arrangement of the local persistency along the network.

Operationally, the generation of the synthetic stream dynamics requires two main steps: the potential network is first defined via a set of nodes with prescribed local persistency; then, a time-variable persistency threshold P*(*t*) is used to discern dry nodes from wet nodes at each time step of the simulation.

### 2.1. Simulating spatial patterns of local persistency

In our framework, the spatial patterns of network dynamics are governed by the spatial distribution of local persistency along the network. These data can be directly estimated from field surveys, as detailed in [38]. However, if empirical data about stream persistency is lacking, as a first-order approximation, the spatial pattern of *P*_*i*_ can be defined exploiting climatic and morphological data. This procedure is divided into three consecutive steps: (i) estimation of the mean network persistency (i.e. the average persistency among all nodes of the network), (ii) definition of the statistical distribution of local persistency, starting from the mean network persistency, and (iii) generation of *N* local persistency values from their statistical distribution and allocation of the resulting *P*_*i*_ values (*i* ∈ (1, *N*)) to all the network nodes.

The first step consists in defining the average persistency along the network, $\bar{P}$ (i.e. the mean network persistency). Previous analyses carried out on a global database encompassing 19 catchments in Europe and the USA have indicated that $\bar{P}$ is mainly controlled by the underlying climatic conditions, with drier sites that are systematically characterized by lower persistencies [14]. Here, to circumvent the need for specifying this key network property through additional independent parameters, we rely on the observed empirical correlation between $\bar{P}$ and the catchment-scale excess precipitation emerging from the observational data of [14], which is summarized by the following empirical equation:

2.1 $$\bar{P}=0.0527+0.1951(P_{t}-E_{p}),$$

where *P*_*t*_ and *E*_*p*_ are, respectively, total precipitation and potential evapotranspiration in mm d^−1^. Equation (2.1) provides a good approximation of the observed values of $\bar{P}$ across several catchments belonging to diverse climatic regions of the world. Due to its inherent empirical nature, however, equation (2.1) might not be applicable to catchments with peculiar geological features—especially if they are located in the most arid or humid regions of the globe. Nonetheless, equation (2.1) allows a useful first-order assessment of the average network persistency of temporary rivers, particularly when empirical data about wet stream dynamics are not available.

The second step defines the cumulative density function of local persistency, *CDF*_*P*_  , which summarizes the spatial variability of local persistency along the ephemeral portion of the river network. Experimental data gathered in a set of catchments located in different geomorphoclimatic regions suggested that the statistical distribution of the local persistency along the river network follows a one-parameter beta probability density function [14]. The corresponding cumulative density function of *P*_*i*_ can be written as follows:

2.2 $$CDF_{P}(P_{i})=1-{\left(1-P_{i}\right)}^{\beta },$$

where $\beta =1/\bar{P}-1$ is inversely related to the average network persistency. Thus, once the mean persistency of a given network is known, or it has been estimated via equation (2.1), the parameter *β* in equation (2.2) can be determined and *CDF*_*P*_ is known. It should be noted that the two empirical relations expressed by equations (2.1) and (2.2) only apply to the temporary portion of the network (i.e. nodes with *P*_*i*_ < 1). Instead, perennial stream portions which never dry out (if any) should be explicitly accounted for by including an atom of probability in *P*_*i*_ = 1 in the *CDF* of the local persistency.

In the third step, each node of the network is assigned a local persistency *P*_*i*_ extracted from the distribution *CDF*_*P*_ , given by equation (2.2). Observed spatial correlations of local persistency in dynamical river networks are typically quite variable across different study sites. While spatial patterns of *P*_*i*_ were found to be positively correlated with some relevant geomorphic properties such as the contributing area [36], in some cases, local heterogeneity in geological features dominates, enhancing the heterogeneity of *P*_*i*_ (e.g. [3,29]). To cope with this issue, here, we identify three different statistically meaningful scenarios. In the first scenario, the local persistency of each node is assigned in a completely random manner, resulting in non-correlated values of *P*_*i*_ along the network. This scenario represents an ideal end-member set-up where the spatial correlation of the nodes’ persistency takes the lowest possible value—probably representative of catchments where the internal heterogeneity of morphometric and geological properties dominates. In the second scenario, instead, *P*_*i*_ is deterministically linked to the topographic wetness index (TWI). The TWI was originally proposed to be a good measure of the likelihood of surface flow in systems with humid climates [39], but it was recently found to be significantly correlated with the observed persistency of the nodes under a variety of climatic settings [29,36,40]. Specifically, quantile transformation (as defined in [41]) is used to estimate the persistency of each node from the corresponding value of TWI. This is done by calculating the empirical *CDF* of TWI along the network, *CDF*_*TWI*_, and then selecting the persistency of each node, *P*_*i*_, such that the equation *CDF*_*TWI*_(*TWI*_*i*_) = *CDF*_*P*_(*P*_*i*_) is fulfilled ∀ *i* (with *CDF*_*P*_ as given by equation (2.2)). As a result, for any pair of nodes *i* and *j*, if *TWI*_*j*_ < *TWI*_*i*_, then *P*_*j*_ < *P*_*i*_. The TWI combines the upstream accumulated area, which monotonically increases downstream, with the local slope. Consequently, while this scenario is characterized by a higher spatial correlation than that observed in the random scenario, we expect *P*_*i*_ to be somewhat heterogeneous, owing to variations of the local slope along the network. Analogously, quantile transformation is used in the third scenario to deterministically link *P*_*i*_ to the upslope accumulated area. The latter is usually proportional to the amount of water supplied from upstream areas and hence is considered a major driver of surface flow occurrence in river networks [30]. In this case, *P*_*i*_ is expected to systematically increase in the downstream direction, thereby guaranteeing the maximization of the spatial correlation of the status of the nodes in the network, for the given *CDF*_*P*_. Consequently, this scenario represents a second end-member set-up, representative of catchments where geomorphic and hydrologic features are spatially uniform. While we recognize the limitations of the statistical approach adopted here, that does not allow the actual prediction of the observed patterns of *P*_*i*_ based on physical attributes of the catchment, we propose that the use of these scenarios represent a parsimonious but reliable strategy to describe the spatial gradients of local persistency in the study sites where such data are unavailable.

### 2.2. Stochastic generation of spatiotemporal dynamics of the active channel network

In this hierarchical framework, the temporal dynamics of expansion and contraction of temporary streams are uniquely determined by the temporal variability of a persistency threshold separating wet from dry nodes, P*(*t*). In the model, the temporal variations of the persistency threshold are driven by the changes in the corresponding streamflow at the outlet, *Q*(*t*). When *Q* increases, P* decreases and the network expands, as more nodes get activated because their local persistency exceeds P*. Conversely, when *Q* decreases, P* increases and the network contracts as more and more nodes get deactivated. When available, time series of observed or modelled *Q*(*t*) can be directly employed for reconstructing the past dynamics of the active network. Alternatively, the physically based stochastic model presented later in this article can be employed to generate synthetic time series of *Q*(*t*) that mirror the natural intermittency of precipitation. The main advantage of the stochastic model used hereafter is that it only requires three parameters with clear physical meaning (namely, effective rainfall frequency, mean daily rainfall depth and recession coefficient), thus enabling a parsimonious generation of long-term scenarios in which the stochastic nature of rainfall is explicitly accounted for.

This model, first introduced by [42,43], has been used several times in literature owing to its robustness and flexibility. The model showed a good capacity of accurately describing flow regimes over a wide array of settings, also in the absence of discharge observations [44]. Rainfall is modelled as a marked Poisson process of mean rate *λ*_*P*_ and exponentially distributed depths with average *α*. Precipitation events infiltrate in the root zone, where they replenish the moisture deficit created by evapotranspiration in between rain events. The drainage towards the stream is assumed to take place only when soil moisture, in response to some rain events, exceeds a given wetness threshold. These fill-and-spill dynamics create a second stochastic process corresponding to the sequence of effective rainfall pulses, resulting from the filtering operated by soil moisture dynamics on the total precipitation. The effective precipitation is modelled as a new Poisson process with mean depth equal to *α* and a frequency *λ*, which is smaller than the precipitation frequency *λ*_*P*_. Effective rainfall is released towards the stream following a linear storage–discharge relation. This results in a sequence of stochastic streamflow increments and exponential recessions at the outlet, and the corresponding dynamics of the catchment-scale discharge *Q* are described by the following equation:

2.3 $$\frac{dQ}{dt}=-kQ+\xi (t),$$

where *k* is the recession rate and *ξ*(*t*) represents the sequence of streamflow increments induced by effective rainfall events. As we shall see later on, the link between *Q* and the corresponding P* crucially determines the portions of the network that are active for any given value of streamflow at the outlet. The cumulative density function of the streamflows resulting from the process described by equation (2.3) can be written as follows:

2.4 $$CDF_{Q}(Q)=\frac{\gamma (\lambda /k,Q/\alpha k)}{\Gamma (\lambda /k)},$$

where Γ(⋅) and *γ*( · , · ) are, respectively, the complete gamma function and the lower incomplete gamma function. The function *CDF*_*Q*_ can be interpreted in terms of the flow duration curve (FDC), which links each streamflow value *Q* to the corresponding relative duration *D*, i.e. the fraction of time for which that specific *Q* is equalled or exceeded. Accordingly, the flow duration curve can be written as follows:

2.5 $$D(Q)=FDC(Q)=1-CDF_{Q}(Q).$$

The dynamics of the active network mimic the sequence of streamflow pulses and recessions observed at the catchment outlet. Owing to the hierarchical structuring of temporary streams, a finite set of network configurations is observed during network expansion, in which less and less persistent nodes progressively activate. In particular, when the network expands, some nodes which were previously dry activate, while all the nodes that were already active remain wet. The same sequence of network configurations is then observed with a reverse order during network contractions. The synchronicity between increases (decreases) of *Q* and network expansion (contraction) implies the existence of a bijective correspondence between *Q* at the outlet and the spatial configuration of the active nodes in the network. This complies with the one-to-one relation between *Q* and active length *L* frequently observed in the literature [19,30,34,40,45,46]. Under the aforementioned assumptions, whenever a streamflow *Q* with duration *D* is observed at the outlet, the corresponding active network configuration is made by nodes that are active for a fraction of time which is at least equal to *D*, because they are active also in all the more expanded configurations observed during higher flow levels. To state it differently, whenever a given network configuration is observed, the corresponding active nodes have a local persistency *P*_*i*_, which is at least equal to the duration *D* of the corresponding length and catchment discharge observed at that time [47],

2.6 $$P^{*}(t)=D(Q(t))=1-CDF_{Q}(Q(t)),$$

where P*(*t*) represents the instantaneous value of persistency threshold that separates active and dry nodes. P*(*t*) can thus be derived based on *Q*(*t*) as simulated via equation (2.3). For each time step, the status of each node of the network is assigned as follows:

2.7 $$X_{i}(t)=\left\{ \begin{array}{cc} 1\; & \text{if}\;P_{i}\geq P^{*}(t)\;\\ 0\; & \text{otherwise}\; \end{array}\right..$$

The length of the active network, *L*(*t*), can then be calculated as the sum of the lengths Δ*l*_*i*_ associated to each active node [38],

2.8 $$L(t)=\sum_{i=0}^{N}\Delta l_{i}\cdot X_{i}(t).$$

It is worth noting that, based on the aforementioned definition, *L* comprises all the active portions of the network, regardless of their degree of connectivity to the outlet (or lack thereof). Moreover, while the scheme originates a one-to-one relationship between the total active length, *L*, and the catchment discharge, *Q* (in line with the existing literature), the shape of this *L*(*Q*) relation is not specified *a priori* but emerges from the model parameters. The maximum length of the stream network is achieved when all the nodes in the network are simultaneously active and is represented by the geomorphic length *L*_*g*_. An effective way to summarize the dynamics of *L*(*t*) is provided by the stream length duration curve (SLDC), which relates each possible active length of the network (*L*) with the corresponding duration *D*. Once *L*(*t*) is known, the SLDC can be easily obtained with the Weibull plotting position method. However, as established by [38], the SLDC can also be directly achieved from the spatial distribution of the local persistency. In particular, 1 − *CDF*_*P*_(P*) can be interpreted as the fraction of nodes with local persistency greater than P*, which also corresponds to the fraction of length (*L*/*L*_*g*_) that is flowing when only all the nodes with *P*_*i*_ ≥ P* are active. Given that P* represents the duration for which said nodes are active, the corresponding length must have a duration *D* = P*. Therefore, the SLDC can be written as follows:

2.9 $$\frac{L}{L_{g}}=1-CDF_{P}(D).$$

This set-up allowed us to generate a stochastic time series of streamflow following equation (2.3), which was transformed into a time series of persistency thresholds with equation (2.6). Finally, starting from the potential network generated in 2.1, equation (2.7) allowed the estimation of the active nodes at each time step. The full spatial and temporal dynamics of a dynamic channel network were thus obtained.

### 2.3. Numerical set-up

Without the loss of generality, the stochastic model presented in this article was applied to a representative stream network derived from a mesoscale catchment in the Italian subalpine region. In particular, a coherent stream map and a digital terrain model publicly available in the Veneto Region Geoportal [48] were used to define the reference geometry of the geomorphic network via 1215 nodes and calculate the associated topographic features (e.g. slope, contributing area). However, all the relevant spatial quantities were made dimensionless in §3 owing to the fractal nature of river networks [49], which makes the spatial scale of the catchment irrelevant for the hydrological analyses performed in this article whenever the precipitation input can be assumed to be spatially uniform.

Two different sets of basic hydroclimatic parameters (*α*, *λ*_*P*_ and *E*_*p*_) were used to simulate two contrasting climatic scenarios, namely, Dry (*D*) and Wet (*W*), as shown in table 1. While these scenarios were selected to explore the system’s behaviour under a gradient of climatic conditions, the ‘Dry’ and ‘Wet’ terms are not directly related to any standard climatic classification, the only real meaning being that the ‘Wet’ scenario is wetter than the ‘Dry’. Nonetheless, the ‘Dry’ scenario can be thought of as representative of the rainy season in a hot Mediterranean climate with high *ET* and sparse but intense rain inputs, while the ‘Wet’ scenario can be seen as representative of the summer season of humid Alpine areas with frequent rainfall events and moderate *ET*. These scenarios were characterized by different values of the parameters *α* and *λ* of the streamflow model, while the recession rate *k* was assumed to be constant and equal to 0.35 d^−1^ [43]. The value of *λ* associated with each scenario was calculated based on the corresponding *λ*_*p*_ and *E*_*p*_, as detailed later in this article. First, the actual evapotranspiration *E*_*a*_ was calculated with the Budyko curve [50,51] as follows:

2.10 $$\frac{E_{a}}{P_{t}}={\left[\frac{E_{p}}{P_{t}}\;\tanh\left(\frac{P_{t}}{E_{p}}\right)\left(1-\exp\left(-\frac{E_{p}}{P_{t}}\right)\right)\right]}^{1/2},$$

where *P*_*t*_ = *αλ*_*P*_ is the mean total precipitation. Subsequently, the mean effective rainfall frequency *λ* was estimated based on the catchment water balance equation proposed by [52],

2.11 $$\lambda =\lambda_{P}-\frac{E_{a}}{\alpha },$$

which expresses the fact that the sequence of effective precipitation pulses is composed of the subset of rainfall events bringing enough water to fill the soil water deficit created by evapotranspiration. Therefore, the mean effective precipitation (*αλ*) can be calculated by subtracting *E*_*a*_ from the mean total precipitation, *P*_*t*_.

**Table 1.** Summary of the parameters used for the simulation of network dynamics in the different climatic scenarios. The recession rate *k* is constant across all scenarios and equal to 0.35 d^−1^. Only *α*, *λ*_*P*_, *E*_*p*_ and *k* are independent parameters.

climatic scenario		dry (*D*)	wet (*W*)	
mean daily rainfall depth	*α*	15.0	10.0	mm d^−1^
mean rainfall frequency	*λ*_*P*_	0.25	0.55	d^−1^
potential evapotranspiration	*E*_*p*_	3.0	2.0	mm d^−1^
mean total precipitation	*P*_*t*_	3.75	5.50	mm d^−1^
actual evapotranspiration	*E*_*a*_	2.29	1.82	mm d^−1^
effective rainfall frequency	*λ*	0.10	0.37	d^−1^
rain freq. to recession rate ratio	*λ*/*k*	0.65	2.45	d^−1^
mean network persistency	$\bar{P}$	0.20	0.74	—

The mean network persistency was then estimated by means of equation (2.1). Local persistency values for each node of the network were randomly extracted from the probability density function shown in equation (2.2). In particular, three different criteria for assigning local persistencies were compared: (1) completely random, resulting in non-correlated persistency along the network, (2) by TWI (i.e. nodes with higher TWI are given higher persistency by means of quantile transformation), and (3) by contributing area. Overall, six scenarios were explored in this article, as determined by the combination of three persistency spatial patterns (1–3) and two different climates (*D* or *W*). These six scenarios are identified by unique two-digit labels indicating the underlying set-up (e.g. the scenario with wet climate and local persistencies defined based on the contributing area was labelled as *W3*).

For each scenario, a 100-year daily time series of catchment streamflow *Q*(*t*) was stochastically generated. The corresponding flow duration curve was then derived and used to generate the time series of persistency threshold P*(*t*) as per §2.2. The status of each node was assigned for each time step by means of equation (2.7), and the active length calculated with equation (2.8). Finally, a Weibull plotting position method was used to construct the SLDC from *L*(*t*), to be compared with equation (2.9). Furthermore, the length of the largest continuous portion (LCP) of the active network (i.e. the sum of the length of the active segments that are connected to each other within the largest patch of the network) was calculated for each possible network configuration. The resulting relation between P* and LCP length was used to summarize the combined effect of the dynamics of active length and the presence of disconnections as the network goes from completely dry (P* = 1) to fully expanded (P* = 0). The LCP length versus P* curve can also be interpreted as a duration curve because P* can be thought of as the duration of the corresponding network configuration.

## 3. Results

### 3.1. Space-time dynamics of the actively flowing stream network

**Figure 1.**
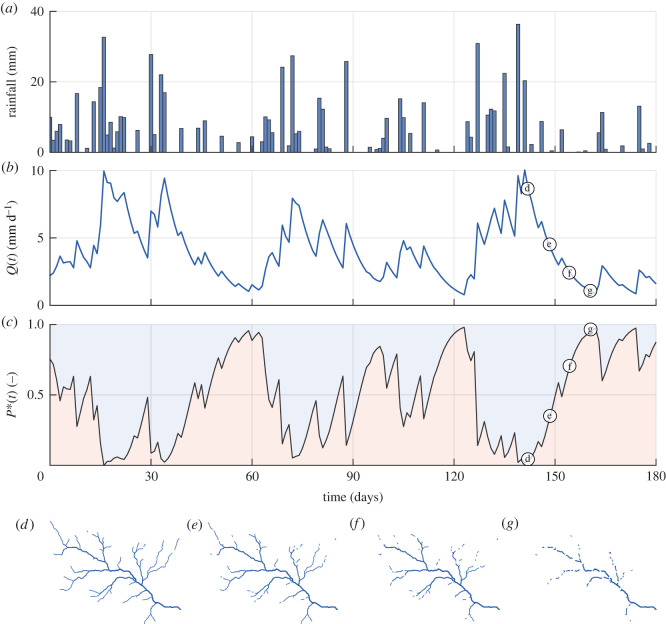
Simulation results for the *W*2 scenario. (*a*)–(*c*) The time series of rainfall, streamflow *Q*(*t*) and persistency threshold P*(*t*). (*d*–*g*) The simulated maps of the active network on the four different time steps indicated in (*c*) and (*d*).

For the sake of illustration, we first analyse in figure 1 the results pertaining to the *W2* scenario. The time series of rainfall (figure 1*a*) mirrored the high frequency of precipitation typical of wet scenarios (*λ* = 0.37 in this case) that guarantees a regular water supply to the catchment storage, often originating sequences of consecutive wet days. The stochasticity of the effective rainfall was directly reflected into the time series of streamflow *Q*(*t*), in which the positive increments in correspondence of the major precipitation events were followed by exponential recessions. The time series of persistency threshold P*(*t*) showed an analogous, albeit reversed, behaviour. In particular, each rainfall event determined an increase of *Q*(*t*), which was reflected into a reduction of P*(*t*) and led to the expansion of the active network. As per equation (2.6), *CDF*_*Q*_ modulated the transformation of *Q*(*t*) into P*(*t*) and introduced a nonlinear relation between these two signals (see also figure 4*c*, in which the FDC also represents equation (2.6) because the duration axis can be reinterpreted as P*(*t*)). In particular, for the *W2* scenario, the variability of *Q* in the range of the highest flows (*Q*(*t*) > 7.5 mm d^−1^) corresponded to weak variations of P*(*t*) (figure 1). Instead, streamflow variations in the range between 2.5 and 5 mm d^−1^ were amplified by P*(*t*), suggesting that the active network length may be most sensitive to the underlying hydroclimatic variability for intermediate flow conditions ($\bar{Q}=4\;{\mathrm{mm}\;d}^{-1}$ for scenario *W2*). Figure 1(*d*–*g*) shows a sequence of snapshots of the simulated active network, while the full dynamics are provided as a video in the electronic supplementary material. The maximum extension of the network (panel *d*) was obtained at day 16 of the simulation, after a series of intense rain events that resulted in a significant increase of catchment discharge. During periods with limited rainfall inputs (e.g. from day 45 to 60), or in the absence of precipitation (e.g. in the period between day 111 and 123), streamflow markedly receded with a progressive contraction of the active network (as shown in figure 1*e*,*f*). The most contracted network configuration was reached after the longest dry spell (figure 1*g*).

**Figure 2.**
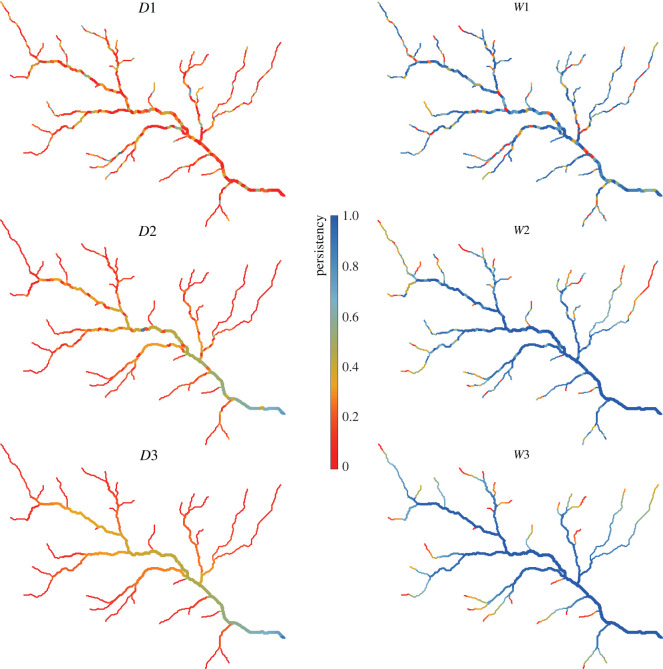
Maps of local persistency for the six simulated scenarios (wet and dry conditions).

**Figure 3.**
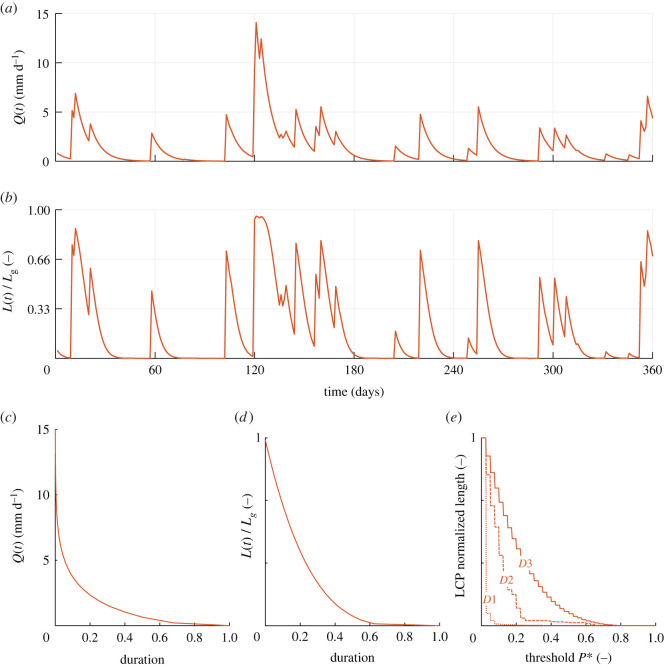
Time series of simulated streamflow (*a*) and active length (*b*) for the dry scenarios. (*c*,*d*) The corresponding flow and stream length duration curves. (*e*) The length of the LCP of the active network, as a function of P*, for the three scenarios of local persistency described in the text. All the results concerning the active length are shown in terms of the relative length, defined as *L*(*t*)/*L*_*g*_, were *L*_*g*_ is the geomorphic length (i.e. the maximum length of the active network when all the nodes are active). This choice emphasizes the fact that the spatial scale of the problem is not relevant for the generation of stream dynamics in the proposed framework.

**Figure 4.**
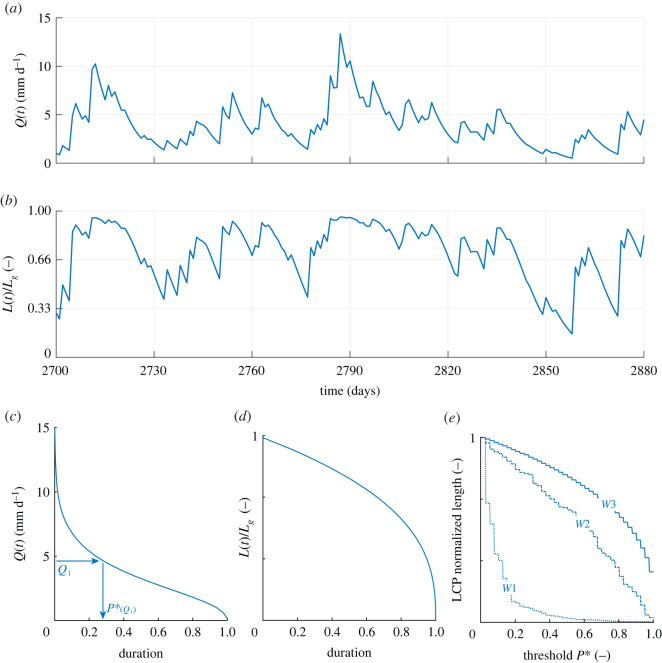
Time series of simulated streamflow (*a*) and active length (*b*) for the wet scenarios. (*c*,*d*) The corresponding flow and stream length duration curves. (*e*) The length of the LCP of active length, as a function of P*, for scenarios *W*1–*W*3.

**Figure 5.**
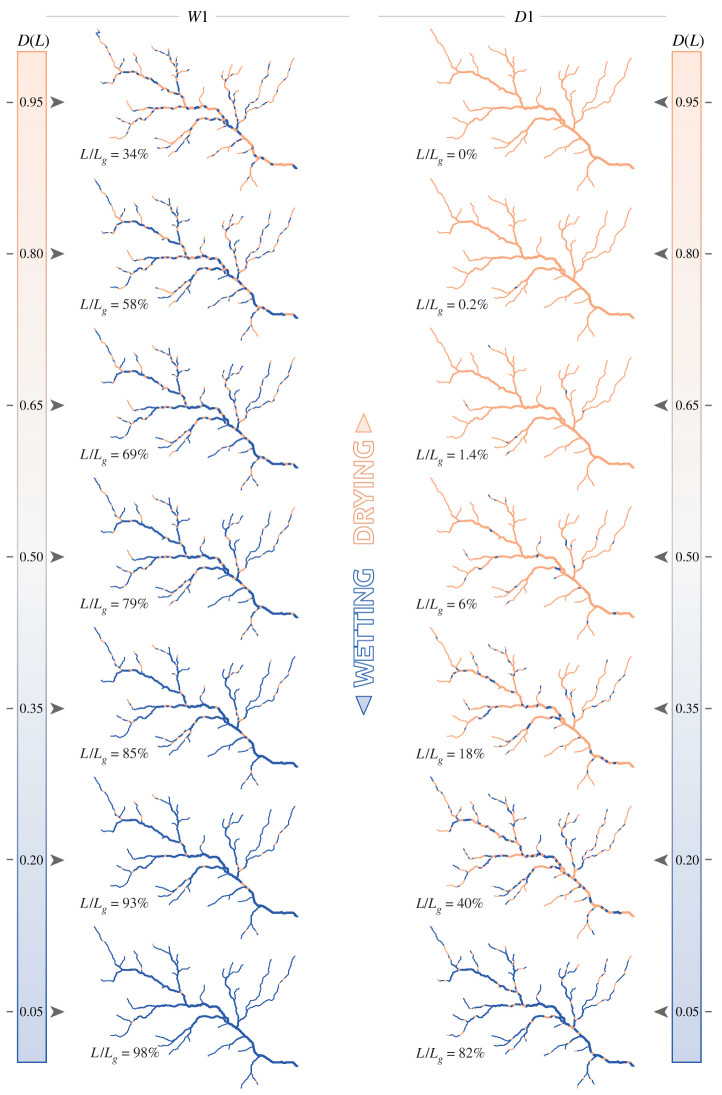
Snapshots of the active network (in blue) for scenarios *D*1 and *W*1, i.e. dry and wet scenarios with random assignment of local persistency, with the corresponding active length and its duration.

**Figure 6.**
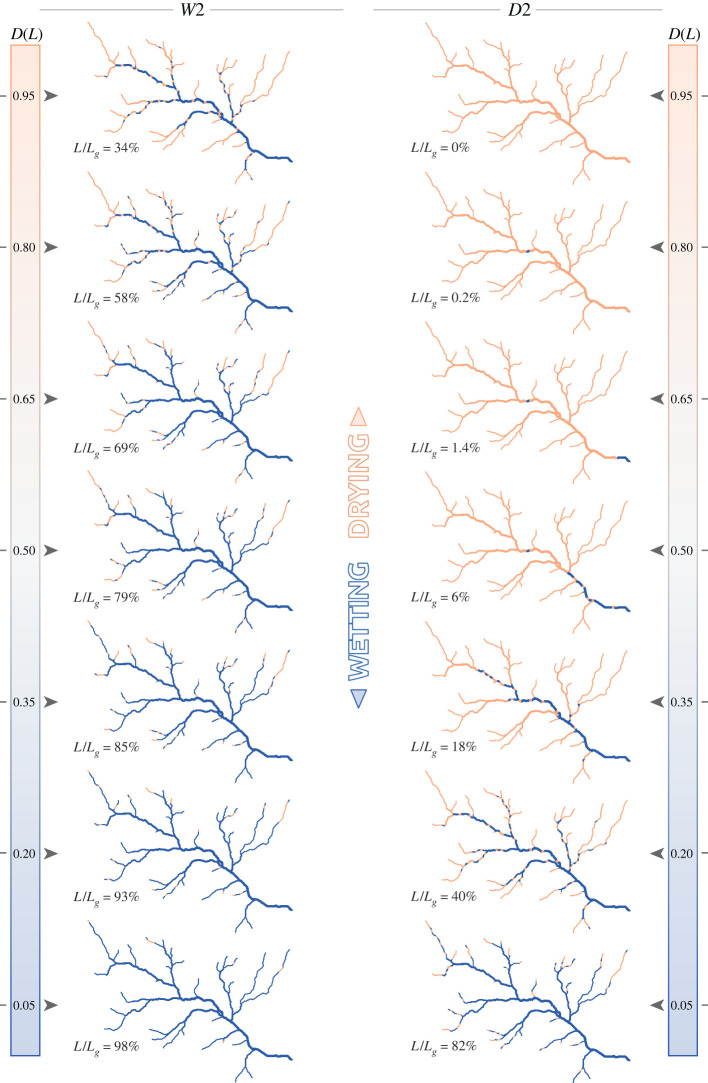
Snapshots of the active network (in blue) for scenarios *D*2 and *W*2, i.e. with local persistencies assigned by TWI, with the corresponding active length and its duration.

**Figure 7.**
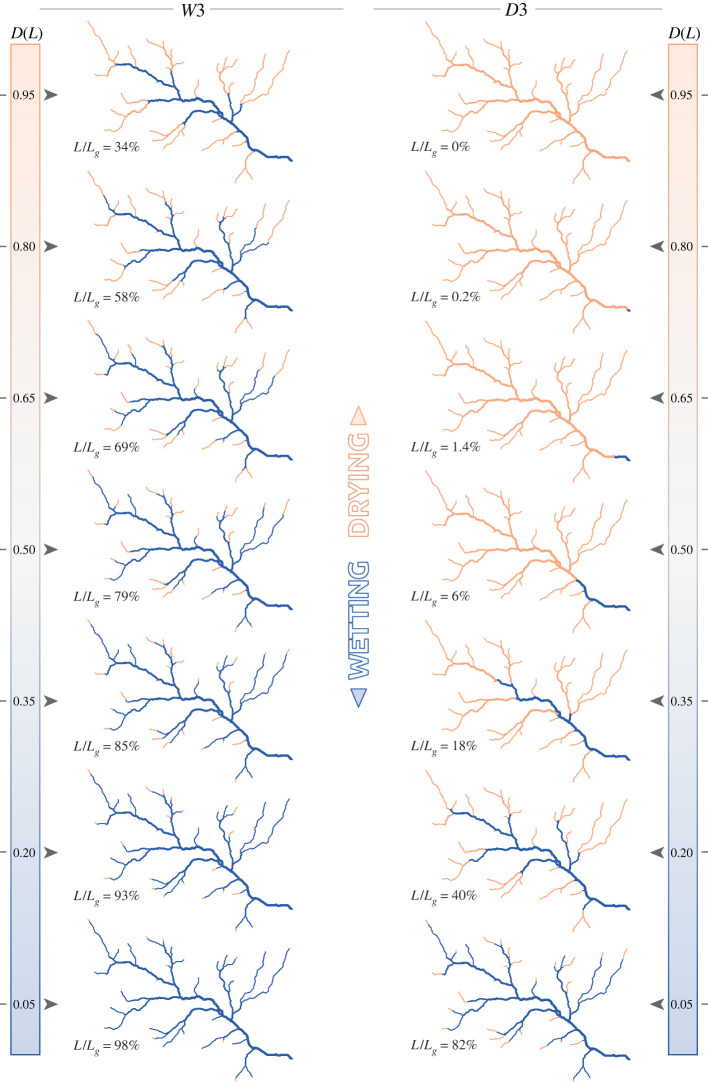
Snapshots of the active network (in blue) for scenarios *D*3 and *W*3, i.e. with local persistencies assigned by contributing area, with the corresponding active length and its duration.

The different dynamics that the river network experienced under the scenarios analysed in the article were explored by comparing the corresponding model outputs: (i) the maps of local persistency (figure 2); (ii) the generated time series of *Q* and *L* (figures 3 and 4) and (iii) the different sequences of possible configurations that each network experiences (figures 5–7). The generated maps of 423 local persistency for the six scenarios corresponding to the wet and dry climates are reported in figure 2. All the dry scenarios (*D1, D2* and D3) shared the same mean persistency $\bar{P}=0.20$ and therefore the same statistical distribution of *P*_*i*_, with the only difference being the underlying spatial patterns. The same applies to the three wet scenarios (*W*1*, W*2 and *W3*), which all shared a mean persistency $\bar{P}$ of 0.74. As expected, randomly assigning the local persistencies (*D*1 and *W*1) generated maps where adjacent nodes might have very different values of *P*_*i*_, whereas if persistencies were assigned based on the underlying contributing area (*D*3 and *W*3), *P*_*i*_ monotonically increased downstream. When local persistencies were assigned based on the TWI (*D*2*, W*2), instead, *P*_*i*_ generally increased in the downstream direction, with a few notable exceptions.

An example time series of streamflows for the dry scenarios is depicted in figure 3*a*. Under the aforementioned circumstances, the recession rate was higher than the mean frequency of flow-producing rainfall events (*λ*/*k* = 0.65), resulting in an erratic streamflow regime where few significant flow pulses were followed by prolonged recessions and low flows. This was reflected by a convex FDC, owing to the high probability associated to very low flows (figure 3*c*). The FDC, being related to *CDF*_*Q*_, determines how *Q*(*t*) modulates the expansion and contraction of the active network. The convex shape of the FDC thus suggested that, under the dry scenarios, P*(*t*) was more sensitive to low flows, for which the FDC was flatter. The corresponding active length time series, shown in figure 3*b*, inherited some of the properties of *Q*(*t*), with a few key differences. The steeper recessions exhibited by active length dynamics compared with the corresponding streamflows generated long spells during which the stream network was almost completely dry. Nonetheless, a remarkable fraction of the network was activated only in response to a few moderate-to-high discharge events. This produced a significant variability in the active length (*CV*_*L*_ = 1.35). The SLDC (figure 3*d*) displayed similar characteristics to the FDC and approached the horizontal axis for a duration of about 0.65. Thus, the network was almost completely dry during about one third of the simulation.

The simulated time series of streamflow and active length for the wet scenarios, as well as the corresponding flow and SLDCs, are reported in figure 4. In this case, the mean interarrival between effective rainfall events was smaller than the recession rate (*λ*/*k* = 2.45), leading to a persistent flow regime with streamflows that were weakly variable around the mean. As a consequence, most of the stream network was active for significant periods of time, with sensible reductions of *L*(*t*) only during the longest recession periods. The active length time series showed how active length recessions were concave shaped in this case, with the active length signal *L*(*t*) that remained quite constant for a few days before starting the contraction stage. This tended to reduce active length variability (compared with the streamflows), resulting in a much lower value of the coefficient of variation of the flowing length, *CV*_*L*_ (*CV*_*L*_ = 0.28). The FDC was knee shaped, suggesting that streamflow fluctuated around its average value, rather than increasing significantly only in response to the most intense rainfall events. The SLDC, instead, was concave and much flatter than that observed under the dry scenario for almost all the durations, in line with the reduced variability of the active length observed in this case. Furthermore, durations *D* = 1 corresponded to a relative length *L*/*L*_*g*_ of about 0.2, revealing that in this case a significant portion of the network remained permanently active.

Figure 5 shows two sets of network configurations associated with durations ranging from 0.05 to 0.95, for the scenarios *W*1 and *D*1 (wet and dry scenarios with random assignment of local persistency). As *D*(*L*) can also be interpreted as the persistency threshold P*, the corresponding expansion/contraction cycles of the active network consist of a continuous sequence of configurations belonging to the set of active network maps reported in the figure. The random assignment of local persistency along the network resulted in a patchy activation of network segments, generating a multitude of disconnections that were gradually removed as the network approached its fully expanded configuration. Furthermore, under the dry scenario (*D*1), owing to the erratic flow regime, most parts of the network were activated only sporadically, and the active network associated with a relative length of 50% had a duration *D* smaller than 0.2. The same network configuration, on the contrary, had a duration *D* > 0.8 under the wet scenario (*W*1). In this case, 93% of the network was active for at least 20% of the time. Nonetheless, multiple disconnections were observed throughout all the possible network configurations (with the only exception of the fully expanded network). Such disconnections were not observed in the *D*3 and *W*3 scenarios, instead, where *P*_*i*_ was proportional to increasing contributing area (figure 7). In this case, the network expanded upstream during wetting, thereby ensuring the full connectivity of its active portion. When the topographic wetness index was used as a criterion for the assignment of *P*_*i*_ (scenarios *D*2 and *W*2 shown in figure 6), the spatial pattern of flowing network combined the characteristics described earlier for the two end-member scenarios. In this case, in fact, network expansion was associated with both the activation of disconnected reaches in the upper part and the removal of disconnections in the downstream portion of the network. As a consequence, the active network was composed by a main part, comprising most of the active length and usually connected to the outlet, plus a number of small disconnected reaches in the uppermost branches of the geomorphic network.

The relation between P* and the length of the LCP of the active network for each of the six hydroclimatic scenarios was also analysed to quantify the impact of stream contraction on the longitudinal connectivity of the network. When local persistencies were assigned according to the contributing area, the networks were fully connected and the LCP curves resembled the corresponding SLDCs, as shown in figures 4*e* and 3*e* for scenarios *W*3 and *D*3, respectively. In scenarios *W*2 and *D*2, where local persistency was assigned by TWI, a number of disconnections arose along the network, and there were several small active branches disconnected from the outlet, thus generating slightly lower values of LCP length. In the case of scenarios *W*1 and *D*1, where the local persistencies were spatially uncorrelated, significant sizes of the LCP occurred only for the lowest values of P* even in the case of wet climatic conditions (figure 4*e*), suggesting that the active network remains fragmented into a multitude of small portions (LCP normalized length<0.1) even for quite expanded network configurations (*L*/*L*_*g*_ ≈ 0.95). As climate varied from wet to dry, the curves move to the left (figure 3*d*), showing how continuous portions of active network with significant sizes are less frequent in ephemeral streams of drier regions.

## 4. Discussion

The hierarchical approach of the proposed model allowed the separation of the temporal and spatial components of network dynamics. The hierarchical structuring of river networks can be interpreted as follows: the time-variable threshold P*(*t*) mimics a highly correlated hydrological signal that is propagated along the network as catchment wetness (and the corresponding streamflow at the outlet) increases and the network expands, while the spatial patterns of local persistency are related to the sensitivity of each part of the network to such hydrological signal. While the physical mechanisms that shape the spatial patterns of local persistency are not explicitly included in the formulation, the main advantage of the proposed model is that it only exploits above-the-ground physical quantities (e.g. precipitation, streamflow) that are easier to measure compared with other relevant subsurface characteristics (e.g. soil hydraulic transmissivity, geological features of the contributing catchments), thereby facilitating the practical application to real-world catchments. However, for this reason, the model may not be able to capture stream dynamics in the cases where the heterogeneity of subsurface is the major driver of the streamflow persistency along the network. These cases would require the development of ad hoc site-specific procedures for assigning the patterns of local persistency.

The approach proposed in this article is new and represents the first stochastic model for simulating the event-based spatiotemporal dynamics of active streams. However, specific parts of the framework have already been published, validated and applied individually. In particular, the framework combines the stochastic model for streamflow generation first introduced by [52] with the hierarchical behaviour of network dynamics established by [14,38]. The coupling is allowed by the one-to-one relation between streamflow and active length, which was commonly observed in many practical settings (e.g. [30,40,45,46]). One of the main advantages of the model is the explicit description of the link between climate, discharge and stream network dynamics. This link unveils how the stochasticity of rainfall is reflected into the expansion/contraction cycles of the active portion of a river network, providing a basis for exploring the ecological implications of these dynamics, as discussed in a companion paper.

Another strength of the proposed framework for the simulation of river network dynamics is the modularity of the approach, and the consequent flexibility in terms of data requirements: while the minimum requirement consists of four basic hydroclimatic parameters (*α*, *λ*_*P*_, *E*_*p*_ and *k*), these could be easily replaced or integrated by other types of data and/or models. For example, the local persistencies of the network nodes, which are here estimated via an empirical relationship that links the mean network persistency to the underlying climate, could instead be directly derived from field observations. Also, available streamflow datasets or different hydrological models for streamflow generation could be employed, with different requirements in terms of parameters and calibration data. This represents a sharp contrast with currently available spatially distributed models for the simulation of network dynamics (e.g. [35]). Physically based formulations, in fact, have a rigid structure that usually requires a lot of parameters and cannot be easily adapted to applications characterized by limited data availability. As a consequence, we propose that this model could become a useful tool, either to construct long-term stochastic scenarios (e.g. for the assessment of climate change effects on temporary streams) or for the simulation of past network dynamics in selected case studies.

The main shortcoming of the presented model lies in the lumped approach used for generating the temporal variability of streamflow and network extent, which empirically summarizes the effect of spatially distributed hydrologic variables such as flow convergence and soil transmissivity. In fact, the physical processes driving the spatial dynamics of the active network are probabilistically described though the variability of the local persistency, which is not mechanistically linked to geomorphic and landscape features. Nonetheless, this model’s characteristic determines very small computational requirements (few seconds needed to simulate the full dynamics of *Q*(*t*), *X*_*i*_(*t*) and *L*(*t*) during 1 year), making it particularly suitable as a basis for integrated, multi-disciplinary modelling exercises, such as the ecological application presented in the companion paper [53].

The model, as presented here, works under the assumption of statistical stationarity, which implies that the average properties of climate does not change through time. Instead, many temporary streams are characterized by seasonal patterns of flow and network dynamics (e.g. [29,54]). Nevertheless, the proposed model could be easily adapted to cope with inter-seasonal variations of mean rainfall and evapotranspiration by adopting different sets of seasonally variable model parameters, as commonly done in stochastic rainfall-runoff models [55].

Our results show that climate jointly influences the temporal variations of streamflow and active length: persistent flow regimes, characteristic of wet scenarios with frequent precipitation (*λ*/*k* > 1) and sustain river networks with limited temporal variations. Therein, the active length variability is damped compared with the underlying discharge fluctuations, which are in turn quite limited. In these scenarios, the active network maintains an expanded configuration for a certain period of time after each discharge event, before the contraction phase is observed. The relatively high precipitation frequency might prevent network contractions for several days or weeks, thus creating a preferential network configuration that corresponds to the fully expanded river network. On the contrary, erratic flow regimes typical of the driest climates result in generally dry networks that are subjected to flashy activations and very quick contractions. Under the aforementioned circumstances, the pronounced time-variability of the catchment discharge is further amplified by *L*. These synthetic results are consistent with empirical observations [29,40,46] and suggest that climate may also have a direct impact on the sensitivity of active length to changes in catchment wetness, as proposed by [14].

While climate influences the temporal variations of the active length, the spatial configurations of the active network are chiefly driven by the spatial pattern of local persistency. The LCP length varies in time as the network expands and retracts, similarly to the total active length. However, the presence of disconnections along the network has a detrimental effect on the LCP length. When the local persistency is characterized by a low spatial correlation, in particular, multiple disconnections are generated and the LCP length remains quite low even in wet scenarios, where the active network is generally well developed. As the LCP length is a proxy for the longitudinal connectivity of the network, our results suggest that the spatial pattern of local persistency may strongly influence the biogeochemical and ecological functioning of temporary streams (e.g. [56,57]). In spite of the recognized importance of temporal changes of network connectivity within fluvial environments, relatively few studies have explicitly addressed this issue in quantitative eco-hydrological studies (e.g. [58,59]). Therefore, we propose that the stochastic framework described in this article may represent a useful tool for investigating the impact of dynamic connectivity on key ecological processes along river corridors, especially in the light of the limited computational efforts and the low data requirement of the proposed method.

## 5. Conclusion

In this article, we proposed a novel parsimonious model for the numerical simulation of the event-based spatiotemporal dynamics experienced by the active portion of fluvial networks. The model integrates a stochastic streamflow model with the concept of hierarchical structuring of temporary streams, which defines the existence of a unique activation (and deactivation) order of different portions of a river network. The main advantages of the proposed framework are related to the reduced number of parameters, the flexibility in terms of data requirements and the limited computational effort. The model enabled both the reconstruction of observed active network dynamics in specific case studies and the simulation of synthetic long-term scenarios, making it quite versatile and suited to a broad range of applications.

The proposed framework was applied to six scenarios characterized by different hydroclimatic conditions, providing novel insight on how rainfall and evapotranspiration drives stream network dynamics. Effective rainfall stochasticity was found to be directly reflected in the frequency and duration of the expansion/contraction cycles experienced by the active network, on which the dynamics of the flowing length of rivers depends. The underlying hydroclimatic regime also influenced the speed of network retraction and the features of the preferential configuration of the flowing stream network. Wetter climates were characterized by slower changes in the network extent when the discharge recedes, thereby originating active river networks that are weakly variable around the most expanded configuration. On the other hand, temporary streams belonging to dry scenarios exhibited active length contractions that were faster than the corresponding flow recessions, so the preferential configuration of the system was a short network in which only few nodes were active.

The spatial pattern of local persistency determined the temporally variable longitudinal connectivity along the active network. When the local persistency monotonically increased downstream, the resulting network was always connected to the outlet. Configurations with a lower spatial correlation of local persistency, instead, were characterized by multiple disconnections and a reduced longitudinal connectivity, even when the network was relatively expanded and in wet scenarios.

The proposed stochastic framework represents a valuable tool for quantifying the impact of river network dynamics on various in-stream biogeochemical and ecological processes across different spatial and temporal scales, as demonstrated by a companion paper [53], where a dynamic patch occupancy model is coupled to the network model presented here to analyse the impact of temporary stream dynamics on aquatic species under different climatic settings.

## Data Availability

All the data and code presented in this paper are available from the Dryad Digital Repsoitory: https://doi.org/10.5061/dryad.0zpc86709 [60].
